# CTLA-4 A49G GENE POLYMORPHISM IS NOT ASSOCIATED WITH VITILIGO IN SOUTH INDIAN POPULATION

**DOI:** 10.4103/0019-5154.60347

**Published:** 2010

**Authors:** Farha Deeba, Rabbani Syed, Jariya Quareen, MA Waheed, Kaiser Jamil, Hanmanth Rao

**Affiliations:** *From the Department of Genetics, Osmania University, India.*; 1 *From the Department of Mahavir Hospital and Research Center, Hyderabad and School of Biotechnology-MGNIRSA, India.*; 2 *From the Department of Central Research Institute of Unani Medicine, Hyderabad, India.*

**Keywords:** *Autoimmune*, *CTLA-4*, *melanocytes*, *polymorphism*, *vitiligo*

## Abstract

**Background:**

Vitiligo or leukoderma is a chronic skin condition that causes loss of pigment due to destruction of melanocytes, resulting in irregular pale patches of skin. Vitiligo is a polygenic disease and is associated with autoimmunity with an unknown etiology.

**Aims:**

One of the candidate genes which has a strong association with several autoimmune diseases is CTLA-4 gene located in chromosome 2q33 region. We investigated the possible association between CTLA-4 gene polymorphism in exon 1 (A49G) and vitiligo in patients from South India and compared the distribution of this polymorphism to matched control groups.

**Patients and Methods:**

The polymorphism was detected by Polymerase Chain Reaction-Restriction Fragment Length Polymorphism (PCR-RFLP) method in 175 patients and 180 normal, age/ethnicity matched individuals. Consistency of genotype frequencies with the Hardy-Weinberg equilibrium was tested using a χ^2^ test.

**Results:**

There was no significant difference between the genotype (*P* = 0.93) and allele (*P* = 0.615) frequencies of CTLA-4 A49G polymorphism in patients and normal healthy individuals. However there was significant association of the CTLA-4 genotype (*P* = 0.02) and allelic frequency (*P* = 0.008) between the segmental and non-segmental sub groups within vitiligo.

**Conclusion:**

Our results indicate that there is no association between *CTLA-4 A49G* gene polymorphism and vitiligo in southern Indian population.

## Introduction

Vitiligo is an acquired depigmentary disorder characterized by the appearance of white patches resulting from the loss of functional melanocytes[1] and melanin from the skin and is associated with autoimmunity.[2] Vitiligo affects 1-4% of the world's population; irrespective of gender and race. Vitiligo has great cosmetic value. Its prevalence is varying from 0.46 to 8.8% in India.[3] The Gujarat and Rajasthan states have the highest prevalence i.e. around 8.8%. Age at onset is variable and the average age of onset is about 20 years.[4] The etiology of the disease remains unknown and several hypotheses exist to explain the etiology. Autocytotoxic (self-destruct) hypothesis is based on the preferential destruction of melanocytes by toxic intermediates of melanin synthesis, while neural hypothesis suggests that the melanocytes could be damaged by neurochemical mediators released from the nerve endings supported by the association of vitiligo with neurological disorders or with peripheral nerve injury.[5] The inheritance of vitiligo may involve genes associated with the biosynthesis of melanin, a response to oxidative stress, and regulation of autoimmunity.[6] The genetics of vitiligo cannot be explained by simple Mendelian genetics.[7] It is characterized by incomplete penetrance, multiple susceptibility loci and genetic heterogeneity.[8]

A few genes reported to contribute to vitiligo susceptibility are Autoimmune regulator I Gene (AIRE I), Cytotoxic T lymphocyte antigen 4 (CTLA 4), Catalase (CAT), Catechol-o- methyl transferase (COMT), Light molecular weight protein (LMP), transporter associated protein (TAP), Angiotensin converting enzyme gene (ACE), Melanocortin 1 receptor (MC1R), agouti signaling protein (ASIP) genes and Lymphoid protein tyrosine phosphatase (PTPN 22) gene—PTPN.[9] The abnormal immune responses frequently found in vitiligo patients have led to the suggestion that at least in some cases the disease has an autoimmune origin.[10] One of the candidate genes that has a strong association with several autoimmune diseases is CTLA-4 gene located in chromosome 2q33 region.[11–15] Therefore, we investigated the possible association between CTLA-4 gene polymorphism in exon 1 (A49G) and vitiligo in southern Indian patients and compared the distribution of this polymorphism to matched control groups.

## Patients and Methods

### Subjects

In this study, 175 unrelated south Indian vitiligo patients (male: 107, female: 68) were referred to our laboratory by collaborating dermatologist. We included 180 (male: 112, 68: female) age/ethnicity matched unrelated healthy individuals, who were randomly selected. None of the healthy individuals had any evidence of vitiligo and autoimmune diseases or a positive family history of vitiligo and autoimmune diseases. Information including age, sex, occupation, age of onset, type of disease, history of autoimmune diseases, familial history of autoimmune diseases and familial history of vitiligo were collected in the context of a questionnaire from patients. This study was approved by the ethics review of CRIUM committee and all subjects gave informed consent.

### Collection of blood samples and DNA extraction

About 5 ml venous blood samples were obtained from all the cases and controls. Genomic DNA was extracted by salting out method.

### CTLA-4 gene amplification

Polymerase chain reaction (PCR) was used to amplify the desired fragment of CTLA-4 gene using the primers described by Donner *et al*. The primer pairs including 5′- GCT CTA CTT CCT GAA GAC CT-3′ and 5′- AGT CTC ACT CAC CTT TGC AG-3′ were used in the reaction and resulted in a fragment with 162 bp amplicon size. A 50 μl PCR amplification mixture containing 50 ng/μl genomic DNA, 0.38 mM dNTPs, 1.65 mM MgCl_2_, 0.1 U Taq DNA polymerase (Bioserve, India), 0.5 pM of each primers and 1X PCR buffer (20 mM Tris-HCl, pH = 8.4 and 50 mM KCl) was used. The amplification was performed for 30cycles under PCR program: 4 min at 94°C for initial denaturation, denaturation at 94°C for 60s, annealing 60°C for 60s, extension at 72°C for 60s and final extension at 72°C for 10 minutes.

### Detection of alleles

Restriction fragment length polymorphism was used for identification of various alleles in CTLA-4 gene, 10 μl of PCR product was mixed with 1 μl (2u/μl) of BbvI restriction enzyme solution (Fermentas) and incubated at 65°C for 16 hours. DNA fragments were resolved in 4% agarose gel at 80V. The gel was stained with ethidium bromide and the bands visualized in gel doc.

### Statistical analysis

The data were analyzed using sigma stat (ver. 3.5) Differences in the alleles or genotypes frequencies were examined by χ^2^ test. Consistency of genotype frequencies with the Hardy-Weinberg equilibrium was tested using a χ^2^ test on a contingency table of observed vs. expected genotype frequencies in each group.

## Results

Patients' ages ranged from 10 to 69 years (mean 30.9 ± 12.3 years) and the age range of our control group was 11-61 years (mean 29.1 ± 9.7 years). The disease age at onset ranged from 1-66 years (mean 24.1 ± 12.5 years). Out of 175 patients, 51(29.1%) had a positive family history of vitiligo, while only two (1.1%) had an associated autoimmune disease. Involvement of hair (leukotrichia) was observed in 30 (17.1%) out of 175 the patients. The observed clinical subtypes of vitiligo among patients were segmental 75 and non-segmental 100. Frequency and percentage of each genotype in patient and control groups are summarized in Table 1. The frequency of CTLA-4 gene A49G polymorphism was compared in 175 vitiligo patients with 180 age/ethnicity and sex matched controls.

**Table 1:** Demographic characteristics of the cases and controls

	Cases n = 175	Controls n = 180
Average age (yr, mean ± SD) (10-69)	30.9 ± 12.32	29.18 ± 9.7 (11-61)
No. of male/female	107/68	112/78
Onset age (yr, mean ± SD) (1-66)	24 ± 12.5	
Smokers	42	10
Alcoholic	34	4
Duration of disease (yr, mean ± SD) (0.2-33)	6.7 ± 6.8	Nil

There were no significant differences between frequencies of CTLA-4 genotypes in patients and age, ethnicity and sex (*P* = 0.9) matched controls. There were also no significant differences between allelic frequencies in patients (*P* = 0.61) and matched control groups Table 2. No association was found between CTLA-4 exon 1 (A49G) genotypes and gender, and familial history of vitiligo. However, there was significant association of the ctla-0 4 genotype (*P* = 0.02) and allelic frequency (*P* = 0.008) between the segmental and non-segmental sub groups within vitiligo Table 3.

**Table 2:** Genotypic frequencies of CTLA-4 gene A49G polymorphism in patients and matched control groups

Genotype	Patients (%)	Control (%)	χ^2^	*P* value
AA	73 (41.7)	71 (39.4)		
AG	81 (46.3)	86 (47.7)	0.2^a^	0.90
GG	21 (12)	23 (12.7)		
Allelic frequencies				
A	228 (65.1)	228 (63.3)	0.25^b^	0.61
G	122 (34.8)	132 (36.6)		

a = Controls vs. patients using the Chi-square test with 3 × 2 contingency table;

b = Controls vs. patients using the Chi-square test with 2 × 2 contingency table

**Table 3:** Distribution of alleles and genotypes for CTLA-4 polymorphism in sub group vitiligo

Genotype	Non-segmental (%)	segmental (%)	χ^2^	*P* value
				
	N = 100	N = 75		
AA	34 (34)	39 (52)		
AG	50 (50)	31 (41.3)	7.14	0.02
GG	16 (16)	5 (6.6)		
Alleles				
A	118 (59)	109 (72.6)	7.02	0.008
G	82 (41)	41 (27.3)		

a = Controls vs. patients using the Chi-square test with 3 × 2 contingency table; b = Controls vs. patients using the chi-square test with 2 × 2 contingency table

## Discussion

Considered to be the most common pigmentary disorder, vitiligo involves complex interaction of environmental and genetic factors that ultimately contribute to melanocyte destruction, resulting in the characteristic depigmented lesions. In the past few years, studies of the genetic epidemiology of vitiligo have led to the recognition that generalized vitiligo is part of a broader autoimmune disease diathesis. Attempts to identify genes involved in susceptibility to generalized vitiligo have involved gene expression studies, genetic association studies of candidate genes, and genome-wide linkage analyses to discover new genes. The involvement of T cells in the pathogenesis of vitiligo has yet to be clearly defined, although it has been reported that melanocytes are capable of presenting antigenic peptide fragments to well-defined proliferative, cytotoxic T-cell clones in an MHC class II-restricted manner. Our study indicates that there is no association between CTLA-4 A49G polymorphism and vitiligo in southern Indian patients (*P* = 0.93).

In a previous study, Kemp *et al*., investigated the association of a microsatellite polymorphism lying in the 3 × UTR region of the CTLA-4 gene with vitiligo but their results indicated that in the absence of accompanying autoimmune diseases there was no association between the 106 bp allele and vitiligo.[10] Since in our study only 1.1% of the patients had a positive history of autoimmune diseases the comparison of the results with regard to this factor was not possible. In our investigation no correlation was observed between genotypes of patients and disease type, and gender. However, our results are contrary to the frequently reported association of CTLA-4 genotypes with several autoimmune diseases.[17,18] To date, several hypotheses have been proposed to define the etiology of vitiligo, some of which are based on the immunological pathogenesis evidences.[18–20] However, most of these immunological pathogenic reactions can also be a consequence of the disease rather than being a cause.[21] Therefore, it is possible that the autoreactivities observed in vitiligo arise in response to challenges to the immune system by non-immunological mechanisms. Accordingly, an integrated theory has been proposed for the disease in which, vitiligo is considered a primary melanocytorrhagic disorder.[22] On the basis of this theory, autoimmune phenomena are secondary to the detachment of melanocytes[23,24] which can be a consequence of increased expression of tenascin[25] and/or local traumas to the skin.[26]

Our results indicated that in South Indian patients, there was no difference in familial history among male and female patients. There was significant difference between the CTLA-4 genotype (*P* = 0.02) and the allelic frequency (*P* = 0.008) between segmental and non-segmental vitiligo patients. It is worth mentioning that neural hypothesis mostly describes segmental type of vitiligo, while autoimmune hypothesis is commonly related to the generalized vitiligo, Ongenae *et al*.[18] In this regard, it is logical to assume different etiologies in the occurrence of vitiligo in the studied segmental and non-segmental subjects. In contrast to the studies in many other autoimmune diseases, we did not find any association between CTLA-4 exon 1 polymorphism and vitiligo. Since the rediscovery of T reg cells, the immuno-regulatory role of protein in the immune responses and the importance of its deregulation in autoimmune diseases have been well established.[27–30] A large body of information exists on the association of CTLA-4 gene polymorphisms and various autoimmune diseases such as Graves' hyperthyroidism, Addison's disease, etc.[31,32] Lack of association between CTLA-4 gene polymorphism and vitiligo might therefore suggest the involvement of other immune regulatory genes and/or reflect a different nature or multiple etiologies for vitiligo. However, our studies do suggest that non-segmental and segmental vitiligo may have different etiologies.
